# Drug resistance patterns, trends, and risk factors for multidrug resistance of tuberculosis in Wenzhou, China: a ten-year retrospective analysis (2014–2023)

**DOI:** 10.3389/fmed.2025.1611322

**Published:** 2025-07-16

**Authors:** Lianpeng Wu, Dandan Xia, Shuya Xu, Xuefeng Lin, Tingting Peng, Xiangao Jiang

**Affiliations:** ^1^Department of Clinical Laboratory Medicine, Wenzhou Central Hospital, The Ding Li Clinical College of Wenzhou Medical University, Wenzhou, Zhejiang, China; ^2^Key Laboratory of Diagnosis and Treatment of New and Recurrent Infectious Diseases of Wenzhou, Wenzhou Sixth People’s Hospital, Wenzhou, Zhejiang, China; ^3^Department of Tuberculosis Clinic, The Ding Li Clinical College of Wenzhou Medical University, Wenzhou Central Hospital, Wenzhou, Zhejiang, China; ^4^Department of Clinical Laboratory Medicine, Yueqing People’s Hospital, Wenzhou, Zhejiang, China; ^5^Department of Infectious Diseases, The Ding Li Clinical College of Wenzhou Medical University, Wenzhou Central Hospital, Wenzhou, Zhejiang, China

**Keywords:** tuberculosis, drug resistance, multidrug-resistant tuberculosis, risk factors, Wenzhou

## Abstract

**Objective:**

Tuberculosis (TB), particularly drug-resistant tuberculosis (DR-TB), remains a major public health threat in China. Despite global efforts, multidrug-resistant tuberculosis (MDR-TB) complicates control strategies. Wenzhou, a densely populated coastal city, lacks localized data on TB drug resistance trends. This study analyzes DR-TB patterns (2014–2023) and identifies MDR-TB risk factors to inform targeted interventions.

**Methods:**

A retrospective study included 10,993 TB patients from Wenzhou Central Hospital. Sociodemographic and phenotypic drug susceptibility testing (pDST) data were extracted from the Tuberculosis Information Management System (TBIMS) of the Chinese Center for Disease Control and Prevention (China CDC) and hospital databases. Resistance definitions followed World Health Organization criteria. Trends in resistance rates and risk factors for MDR-TB were evaluated using chi-square tests and multivariate logistic regression.

**Results:**

Among 10,993 patients, 20.41% had DR-TB. Resistance rates in new patients were highest for isoniazid (12.15%) and streptomycin (10.89%), while retreated patients showed higher resistance to isoniazid (34.61%) and rifampicin (27.04%). The overall drug resistance rate of DR-TB decreased from 26.01% (2014) to 19.31% (2023), driven by a decline in retreated cases (64.19%–28.57%), whereas the proportion in new cases remained stable (∼18%). The proportion of MDR-TB in retreated patients fell from 47.30% to 18.37%, but increased slightly in new cases (2.51%–3.86%). Risk factors for MDR-TB included age <65 years (OR = 1.496–1.640), Han ethnicity (OR = 1.911), migrant status (OR = 1.296), unemployment (OR = 1.819), and prior TB treatment (OR = 7.513).

**Conclusion:**

Drug-resistant tuberculosis prevalence in Wenzhou declined over the decade, largely due to improved management of retreated cases. However, persistent primary DR-TB transmission among new patients highlights the need for enhanced active screening and targeted interventions. High-risk groups, including young people, individuals of Han ethnicity, migrants, unemployed individuals, and retreated patients, require prioritized attention in TB control strategies.

## 1 Introduction

Tuberculosis (TB) has resurged as the leading infectious disease killer, posing a significant threat to public health (1, 2). The 2024 Global Tuberculosis Report indicates that in 2023, an estimated 10.8 million individuals worldwide were affected by TB, resulting in approximately 1.25 million fatalities attributed to the disease (3). The World Health Organization has set an ambitious goal of eradicating the TB epidemic by 2035, however, significant challenges remain in achieving this objective (4). Notably, the rise of drug-resistant tuberculosis (DR-TB), particularly multidrug-resistant tuberculosis (MDR-TB), complicates TB prevention and control efforts (5). In 2023, around 400,000 patients globally were diagnosed with Multidrug-resistant/rifampicin-resistant tuberculosis (MDR/RR-TB), with estimates suggesting that 3.2% of new patients and 16% of previously treated patients are afflicted by this form of the disease. In China, the estimated incidence of MDR/RR-TB is 29,000, affecting 2.9% of new cases and 18.7% of previously treated patients (3), highlighting the urgent need to address the DR-TB epidemic in the country. Given China’s vast territory, characterized by diverse land areas, population densities, and varying economic conditions, research conducted by Pan et al. (6) in Dalian, Lan et al. (7) in Guizhou, and Zhang et al. (8) in Chongqing has revealed distinct drug resistance patterns and trends across different regions. Therefore, understanding the local drug resistance patterns and trends among TB patients is essential for effective prevention, control, and treatment of DR-TB. To date, there has been no relevant research focusing on the drug resistance patterns and trends of TB in Wenzhou. This study aims to analyze the drug resistance patterns and trends of TB patients in Wenzhou from 2014 to 2023 and identify the risk factors associated with multidrug resistance, thereby providing valuable insights for the prevention, control of transmission, and treatment of DR-TB.

## 2 Materials and methods

### 2.1 Study setting, population, and data collection

**FIGURE 1:**
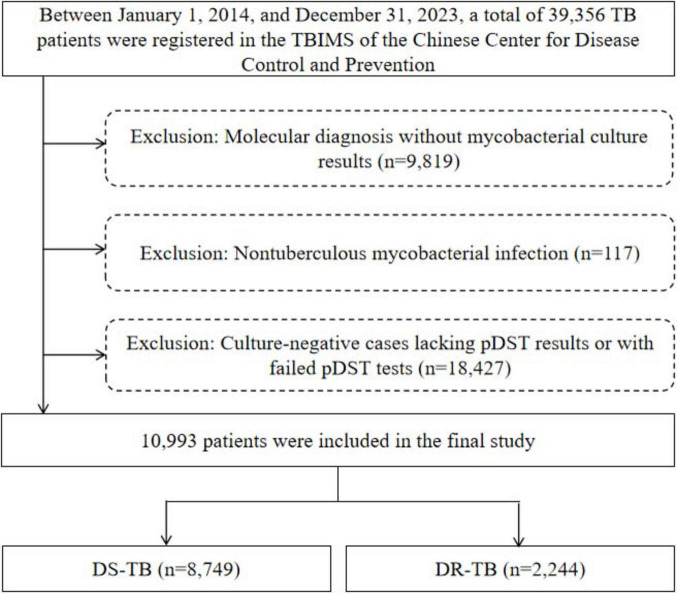
Flow chart of the patients enrolled in the study. TBIMS, tuberculosis information management system; TB, tuberculosis; DS-TB, drug-susceptible tuberculosis; DR-TB, drug-resistant tuberculosis.

This study was conducted in Wenzhou, a coastal city in southeastern China, with geographical coordinates ranging from 119° 37′ to 121° 18′ east longitude and 27° 03′ to 28° 36′ north latitude. The city covers a total area of 12,110 square kilometers, comprising four districts, five counties, and three county-level cities. As of the end of 2023, the permanent population of Wenzhou was approximately 9.761 million, including 4.42 million migrant population. The research was carried out at Wenzhou Central Hospital, a municipal-level medical institution designated for TB treatment. Sociodemographic data and phenotypic drug susceptibility testing (pDST) results of the participants were extracted from the Tuberculosis Information Management System (TBIMS) of the Chinese Center for Disease Control and Prevention (China CDC) and the hospital’s laboratory information system. From January 1, 2014, to December 31, 2023, a total of 39,356 TB cases were registered in the China CDC Information System in Wenzhou. Among them, 9,819 cases relying solely on molecular diagnosis without mycobacterial culture results were excluded. Additionally, 117 cases confirmed as non-tuberculous mycobacterial infections by mycobacterial culture were excluded, along with 18,427 culture-negative cases lacking pDST results or had failed such tests. Ultimately, 10,993 eligible patients were included in the study (see Figure 1).

### 2.2 Definitions

Any drug resistance refers to resistance to a particular anti-TB drug. Mono-resistant tuberculosis (MR-TB) is defined as resistance to one first-line anti-TB drug only. Polydrug-resistant tuberculosis (PDR-TB) is defined as resistance to two or more first-line anti-TB drugs (except both isoniazid and rifampicin). MDR-TB is defined as resistance to at least both isoniazid and rifampicin. New TB cases refer to individuals who have never received TB treatment or have received TB treatment for less than 1 month. Retreated TB cases refer to individuals who have received irregular anti-TB treatment for more than 1 month, as well as those who have failed initial treatment and have relapsed (9).

### 2.3 Drug susceptibility testing

Sputum or bronchoalveolar lavage fluid specimens (3–5 ml) were collected from patients upon admission, pre-treated with NALC-NaOH, and subsequently inoculated into BACTEC MGIT liquid culture tubes or Löwenstein-Jensen (LJ) solid culture medium, then cultured at 37°C. Clinical isolates with positive cultures were confirmed as *Mycobacterium tuberculosis* (MTB) through smear Ziehl-Neelsen staining followed by microscopic examination and MPB64 antigen detection. MTB pDST was conducted using the MGIT liquid method, and all procedures were performed in accordance with the operating instructions for the instruments and reagents. The concentrations of the four first-line anti-tuberculosis drugs (FLDs) were as follows: 1.0 μg/mL for streptomycin (SM), 0.1 μg/mL for isoniazid (INH), 1.0 μg/mL for rifampicin (RFP), and 5.0 μg/mL for ethambutol (EMB).

### 2.4 Statistical analysis

All data were imported into WPS Excel (version 12.1.0.18276, Kingsoft Office Software Co., Ltd., Beijing, China) to create a database, and WPS Excel was also utilized to generate a drug resistance trend chart. Statistical analysis was conducted using SPSS software (version 26.0, IBM, New York, USA). Count data were expressed as frequencies and percentages, while categorical variables were compared using either the Pearson chi-square test or the continuity-corrected chi-square test. The trend chi-square test was employed to analyze the temporal trends in the TB drug resistance model. Univariate logistic analysis was performed on the demographic data of patients, followed by multivariate logistic regression analysis on the variables that exhibited statistical significance in the univariate analysis, in order to identify the risk factors associated with MDR-TB. A significance level of *P*-value < 0.05 was considered statistically significant.

## 3 Results

### 3.1 Patients’ characteristics

From January 1, 2014, to December 31, 2023, a total of 10,993 TB patients were included in the study. This cohort comprised 8,749 patients with Drug-susceptible tuberculosis (DS-TB, 79.59%) and 2,244 patients with DR-TB (20.41%). Within the DR-TB group, there was a higher proportion of males, individuals aged 45–64 years, those of Han ethnicity, residents from rural areas, the unemployed, and patients with a history of TB treatment, compared to the DS-TB group (73.89% vs. 71.69%, 35.52% vs. 31.62%, 97.86% vs. 96.98%, 55.44% vs. 48.18%, 77.05% vs. 72.00%, 20.86% vs. 8.09%). Conversely, the proportion of patients aged under 45 years in the DR-TB group was lower than that in the DS-TB group (46.75% vs. 50.44%). The proportion of migrants was consistent across both groups (43.54% vs. 43.54%) (Table 1).

**TABLE 1:** Sociodemographic characteristics of TB patients.

Characteristics		Total, *n* = 10993 (%)	DS-TB, *n* = 8749 (%)	DR-TB, *n* = 2244 (%)	*P*-value
Gender	Male	7930 (72.14)	6272 (71.69)	1658 (73.89)	0.038
	Female	3063 (27.86)	2477 (28.31)	586 (26.11)	
Age (years)	<45	5462 (49.69)	4413 (50.44)	1049 (46.75)	0.001
	45–64	3563 (32.41)	2766 (31.62)	797 (35.52)	
	>64	1968 (17.90)	1570 (17.94)	398 (17.73)	
Ethnicity	Han	10681 (97.16)	8485 (96.98)	2196 (97.86)	0.025
	Others	312 (2.84)	264 (3.02)	48 (2.14)	
Migrant	Yes	4786 (43.54)	3809 (43.54)	977 (43.54)	0.999
	No	6207 (56.46)	4940 (56.46)	1267 (56.46)	
Residence	Urban	5534 (50.34)	4534 (51.82)	1000 (44.56)	<0.001
	Rural	5459 (49.66)	4215 (48.18)	1244 (55.44)	
Occupation	Employed	2965 (26.97)	2450 (28.00)	515 (22.95)	<0.001
	Unemployed	8028 (73.03)	6299 (72.00)	1729 (77.05)	
Patient category	New cases	9817 (89.30)	8041 (91.91)	1776 (79.14)	<0.001
	Retreated cases	1176 (10.70)	708 (8.09)	468 (20.86)	

DS-TB, drug-susceptible tuberculosis; DR-TB, drug-resistant tuberculosis.

### 3.2 Drug resistance patterns in new and retreated TB patients

Among 9,817 new TB clinical isolates, the drug resistance rates for the four FLDs were as follows: INH (12.15%), SM (10.89%), RFP (5.27%), and EMB (2.71%). In contrast, among 1,176 clinical isolates of retreated TB, the drug resistance rates for the four FLDs were: INH (34.61%), RFP (27.04%), SM (23.47%), and EMB (11.65%). The proportion of DR-TB and MDR-TB in new patients was significantly lower than in retreated patients, with differences that were statistically significant (18.09% vs. 39.79%, 4.42% vs. 25.51%, all *P* < 0.001). The proportion of PDR-TB in new patients was slightly lower than that in retreated patients, although this difference was not statistically significant (3.77% vs. 4.76%, *P* = 0.095). Conversely, the proportion of MR-TB in new patients was slightly higher than that in retreated patients, again with no significant difference (9.90% vs. 9.52%, *P* = 0.682) (Table 2).

**TABLE 2:** Drug resistance patterns in new and retreated TB patients.

Drug resistance pattern	New case, *n* = 9817 (%)	Retreated case, *n* = 1176 (%)	*P*-value
DR-TB	1776 (18.09)	468 (39.79)	<0.001
**Any resistance**			
Any SM	1069 (10.89)	276 (23.47)	<0.001
Any INH	1193 (12.15)	407 (34.61)	<0.001
Any RFP	517 (5.27)	318 (27.04)	<0.001
Any EMB	266 (2.71)	137 (11.65)	<0.001
MR-TB	972 (9.90)	112 (9.52)	0.682
SM	455 (4.63)	40 (3.40)	0.054
INH	416 (4.24)	55 (4.68)	0.482
RFP	58 (0.59)	14 (1.19)	0.016
EMB	43 (0.44)	3 (0.26)	0.497
PDR-TB	370 (3.77)	56 (4.76)	0.095
SM + RFP	7 (0.07)	0 (0)	0.761
SM + INH	302 (3.08)	38 (3.23)	0.772
SM + EMB	2 (0.02)	0 (0)	1.000
RFP + EMB	18 (0.18)	4 (0.34)	0.492
INH + EMB	26 (0.26)	4 (0.34)	0.863
SM + INH + EMB	15 (0.15)	10 (0.85)	<0.001
MDR-TB	434 (4.42)	300 (25.51)	<0.001
INH + RFP	115 (1.17)	83 (7.06)	<0.001
INH + RFP + SM	157 (1.60)	101 (8.59)	<0.001
INH + RFP + EMB	31 (0.32)	29 (2.47)	<0.001
INH + RFP + EMB + SM	131 (1.33)	87 (7.40)	<0.001

DR-TB, drug-resistant tuberculosis; MR-TB, mono-resistant tuberculosis; PDR-TB, polydrug-resistant tuberculosis; MDR-TB, multidrug-resistant tuberculosis; SM, streptomycin; INH, isoniazid; RFP, rifampicin; EMB, ethambutol.

### 3.3 Annual drug resistance trends of TB in Wenzhou from 2014 to 2023

**FIGURE 2:**
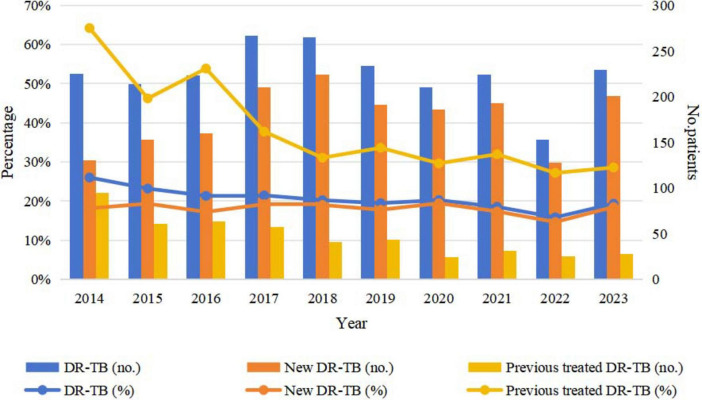
Trends in DR-TB among new and retreated TB patients from 2014 to 2023. TB, tuberculosis; DR-TB, drug-resistant tuberculosis.

The prevalence of DR-TB among all TB patients decreased from 26.01% in 2014 to 19.31% in 2023, reflecting an average annual decline rate of 3.26%, which indicates a significant downward trend (χ^2^ = 39.695, *P* < 0.001). Conversely, the proportion of DR-TB among new TB patients increased from 18.13% in 2014 to 18.47% in 2023, demonstrating an average annual increase rate of 0.21%, thereby indicating a slight upward trend (χ^2^ = 1.514, *P* = 0.219). In contrast, the proportion of DR-TB among retreated patients decreased from 64.19% in 2014 to 28.57% in 2023, exhibiting an average annual decline rate of 8.60%, which signifies a substantial downward trend (χ^2^ = 53.705, *P* < 0.001) (Figure 2 and Table 3).

**TABLE 3:** Annual drug resistance trends of TB in Wenzhou from 2014 to 2023.

Year	Total case, *n* (%)		New case, *n* (%)		Retreated case, *n* (%)	
	No.	DR-TB	No.	DR-TB	No.	DR-TB
2014	865	225 (26.01)	717	130 (18.13)	148	95 (64.19)
2015	923	214 (23.19)	791	153 (19.34)	132	61 (46.21)
2016	1046	223 (21.32)	929	160 (17.22)	117	63 (53.85)
2017	1245	267 (21.45)	1094	210 (19.20)	151	57 (37.75)
2018	1311	265 (20.21)	1179	224 (19.00)	132	41 (31.06)
2019	1203	234 (19.45)	1075	191 (17.77)	128	43 (33.59)
2020	1038	210 (20.23)	957	186 (19.44)	81	24 (29.63)
2021	1210	224 (18.51)	1113	193 (17.34)	97	31 (31.96)
2022	966	153 (15.84)	874	128 (14.65)	92	25 (27.17)
2023	1186	229 (19.31)	1088	201 (18.47)	98	28 (28.57)
χ^2^		39.695		1.514		53.705
*P*-value		<0.001		0.219		<0.001

DR-TB, drug-resistant tuberculosis.

### 3.4 Trends in FLDs resistance rates among new and retreated TB patients from 2014 to 2023

**FIGURE 3:**
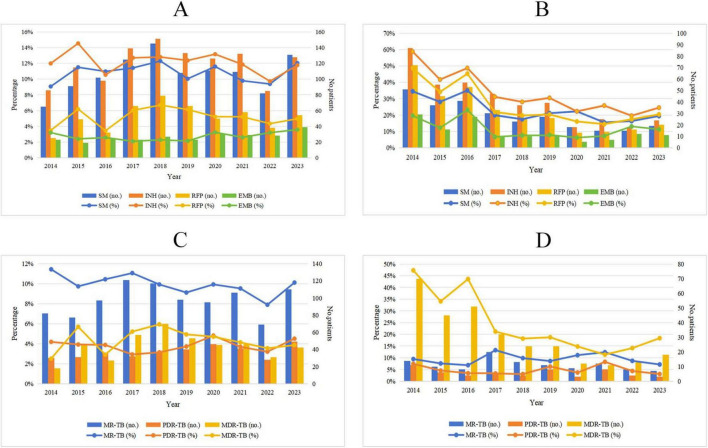
Trends of drug resistance among TB patients in Wenzhou from 2014 to 2023. **(A)** Trends in drug resistance to INH, RIF, EMB and SM among new TB patients; **(B)** trends in drug resistance to INH, RIF, EMB, and SM among retreated TB patients; **(C)** trends in proportions of MR-TB, PDR-TB, and MDR-TB among new TB patients; **(D)** trends in proportions of MR-TB, PDR-TB, and MDR-TB among retreated TB patients. SM, streptomycin; INH, isoniazid; RFP, rifampicin; EMB, ethambutol, MR-TB, mono-resistant tuberculosis; PDR-TB, polydrug-resistant tuberculosis; MDR-TB, multidrug-resistant tuberculosis.

Among new TB patients, the drug resistance rate for SM increased from 9.07% in 2014 to 12.04% in 2023 (χ^2^ = 0.009, *P* = 0.925), while the drug resistance rate for INH decreased from 11.99% in 2014 to 11.76% in 2023 (χ^2^ = 1.883, *P* = 0.170). Additionally, the drug resistance rate for RFP rose from 3.49% in 2014 to 4.96% in 2023 (χ^2^ = 0.043, *P* = 0.836), and the resistance rate for EMB increased from 3.21% in 2014 to 3.58% in 2023 (χ^2^ = 2.316, *P* = 0.128). However, the observed changes in these rates were not statistically significant. In contrast, among retreated TB patients, the resistance rate for SM decreased from 34.36% in 2014 to 19.39% in 2023 (χ^2^ = 19.028, *P* < 0.001), and the resistance rate for INH declined from 58.78% in 2014 to 24.49% in 2023 (χ^2^ = 59.023, *P* < 0.001). Furthermore, the resistance rate for RFP fell from 48.65% in 2014 to 20.41% in 2023 (χ^2^ = 55.700, *P* < 0.001), and the resistance rate for EMB decreased from 19.59% in 2014 to 11.22% in 2023 (χ^2^ = 8.665, *P* = 0.003). Notably, these downward trends were statistically significant (Figure 3 and Table 4).

**TABLE 4:** Trends in FLDs resistance rates among new and retreated TB patients from 2014 to 2023.

Year	New case, *n* (%)					Retreated case, *n* (%)				
	No.	SM	INH	RPF	EMB	No.	SM	INH	RPF	EMB
2014	717	65 (9.07)	86 (11.99)	25 (3.49)	23 (3.21)	148	51 (34.36)	87 (58.78)	72 (48.65)	29 (19.59)
2015	791	91 (11.50)	115 (14.54)	49 (6.19)	19 (2.40)	132	37 (28.03)	55 (41.67)	45 (34.09)	16 (12.12)
2016	929	102 (10.98)	98 (10.55)	32 (3.44)	24 (2.58)	117	41 (35.04)	57 (48.72)	53 (45.30)	27 (23.08)
2017	1094	125 (11.43)	139 (12.71)	66 (6.03)	23 (2.10)	151	30 (19.87)	47 (31.13)	33 (21.85)	10 (6.62)
2018	1179	145 (12.30)	151 (12.81)	79 (6.70)	27 (2.29)	132	23 (17.42)	37 (28.03)	26 (19.70)	10 (7.58)
2019	1075	108 (10.05)	133 (12.37)	66 (6.14)	23 (2.14)	128	27 (21.09)	39 (30.47)	26 (20.31)	10 (7.81)
2020	957	111 (11.60)	126 (13.17)	50 (5.22)	31 (3.24)	81	18 (22.22)	18 (22.22)	13 (16.05)	5 (6.17)
2021	1113	109 (9.79)	132 (11.86)	58 (5.21)	29 (2.61)	97	15 (15.46)	25 (25.77)	14 (14.43)	7 (7.22)
2022	874	82 (9.38)	85 (9.73)	38 (4.35)	28 (3.20)	92	15 (16.30)	18 (19.57)	16 (17.39)	12 (13.04)
2023	1088	131 (12.04)	128 (11.76)	54 (4.96)	39 (3.58)	98	19 (19.39)	24 (24.49)	20 (20.41)	11 (11.22)
χ^2^		0.009	1.883	0.043	2.316		19.028	59.023	55.700	8.665
*P*-value		0.925	0.170	0.836	0.128		<0.001	<0.001	<0.001	0.003

SM, streptomycin; INH, isoniazid; RFP, rifampicin; EMB, ethambutol.

### 3.5 Trends in proportions of MR-TB, PDR-TB, and MDR-TB among new and retreated TB patients from 2014 to 2023

Among new TB patients, the proportion of MR-TB decreased from 11.44% in 2014 to 10.11% in 2023 (χ^2^ = 3.042, *P* = 0.081). Conversely, the proportion of PDR-TB increased from 4.18% in 2014 to 4.50% in 2023 (χ^2^ = 0.301, *P* = 0.583), and the proportion of MDR-TB rose from 2.51% in 2014 to 3.86% in 2023 (χ^2^ = 0.077, *P* = 0.781). However, these changes were not statistically significant. Among retreated TB patients, the proportion of MR-TB declined from 9.46% in 2014 to 7.14% in 2023 (χ^2^ = 0.017, *P* = 0.896), although this downward trend was also not statistically significant. The proportion of PDR-TB decreased from 7.43% in 2014 to 3.06% in 2023 (χ^2^ = 0.196, *P* = 0.658), with this reduction not reaching statistical significance either. In contrast, the proportion of MDR-TB decreased significantly from 47.30% in 2014 to 18.37% in 2023 (χ^2^ = 65.618, *P* < 0.001) (Figure 3 and Table 5).

**TABLE 5:** Trends in proportions of MR-TB, PDR-TB, and MDR-TB among new and retreated TB patients from 2014 to 2023.

Year	New case, *n* (%)				Retreated case, *n* (%)			
	No.	MR-TB	PDR-TB	MDR-TB	No.	MR-TB	PDR-TB	MDR-TB
2014	717	82 (11.44)	30 (4.18)	18 (2.51)	148	14 (9.46)	11 (7.43)	70 (47.30)
2015	791	77 (9.73)	31 (3.92)	45 (5.69)	132	10 (7.58)	6 (4.55)	45 (34.09)
2016	929	97 (10.44)	36 (3.88)	27 (2.91)	117	8 (6.84)	4 (3.42)	51 (43.59)
2017	1094	121 (11.06)	32 (2.93)	57 (5.21)	151	20 (13.25)	5 (3.31)	32 (21.19)
2018	1179	117 (9.92)	37 (3.14)	70 (5.94)	132	13 (9.85)	4 (3.03)	24 (18.18)
2019	1075	98 (9.12)	40 (3.72)	53 (4.93)	128	11 (8.59)	8 (6.25)	24 (18.75)
2020	957	95 (9.93)	46 (4.81)	45 (4.70)	81	9 (11.11)	3 (3.70)	12 (14.81)
2021	1113	106 (9.52)	41 (3.68)	46 (4.13)	97	12 (12.37)	8 (8.25)	11 (11.34)
2022	874	69 (7.89)	28 (3.20)	31 (3.55)	92	8 (8.70)	4 (4.35)	13 (14.13)
2023	1088	110 (10.11)	49 (4.50)	42 (3.86)	98	7 (7.14)	3 (3.06)	18 (18.37)
χ^2^		3.042	0.301	0.077		0.017	0.196	65.618
*P*-value		0.081	0.583	0.781		0.896	0.658	<0.001

MR-TB, mono-resistant tuberculosis; PDR-TB, polydrug-resistant tuberculosis; MDR-TB, multidrug-resistant tuberculosis.

### 3.6 Association between sociodemographic characteristics of TB patients and MDR-TB

Multivariate analysis revealed that the risk of MDR-TB in patients under 45 years of age was 1.640 times higher than that of patients aged 65 and above (OR = 1.640, 95% CI: 1.281–2.099). Additionally, the risk of MDR-TB in patients aged 45–64 years was found to be 1.496 times greater than that of patients aged 65 and older (OR = 1.496, 95% CI: 1.167–1.918). Furthermore, Han patients exhibited a risk of MDR-TB that was 1.911 times that of non-Han patients (OR = 1.911, 95% CI: 1.022–3.572). Migrant patients faced a risk of MDR-TB that was 1.296 times higher than that of non-migrant patients (OR = 1.296, 95% CI: 1.098–1.529). The risk of MDR-TB in unemployed patients was 1.819 times greater than that of employed patients (OR = 1.819, 95% CI: 1.487–2.226). Lastly, patients with a history of previous treatment had a risk of MDR-TB that was 7.513 times higher than that of new patients (OR = 7.513, 95% CI: 6.372–8.859) (Table 6).

**TABLE 6:** Univariable and multivariable logistic regression analyzes of sociodemographic characteristics for MDR-TB among TB patients.

Characteristics		Non-MDR-TB, *N* (%)	MDR-TB, *N* (%)	OR (95% CI)	*P*-value	AOR (95% CI)	*P*-value
Gender	Female	7394 (72.07)	536 (73.02)	1		–	
	Male	2865 (27.93)	198 (26.98)	1.049 (0.886–1.242)	0.579	–	
Age (years)	>64	1868 (18.21)	100 (13.62)	1		1	0.001
	45–64	3301 (32.18)	262 (35.70)	1.483 (1.169–1.880)	0.001	1.496 (1.167–1.918)	
	<45	5090 (49.61)	372 (50.68)	1.365 (1.088–1.713)	0.007	1.640 (1.281–2.099)	<0.001
Ethnicity	Others	301 (2.93)	11 (1.50)	1		1	0.042
	Han	9958 (97.07)	723 (98.50)	1.987 (1.083–3.643)	0.026	1.911 (1.022–3.572)	
Migrant	No	5825 (56.78)	382 (52.04)	1		1	0.002
	Yes	4434 (43.22)	352 (47.96)	1.211 (1.042–1.406)	0.013	1.296 (1.098–1.529)	
Residence	Urban	5191 (50.60)	343 (46.73)	1		1	0.142
	Rural	5068 (49.40)	391 (53.27)	1.168 (1.005–1.357)	0.043	1.125 (0.961–1.317)	
Occupation	Employed	2823 (27.52)	142 (19.35)	1		1	<0.001
	Unemployed	7436 (72.48)	592 (80.65)	1.583 (1.311–1.910)	<0.001	1.819 (1.487–2.226)	
Patient category	New cases	9383 (91.46)	434 (59.13)	1		1	<0.001
	Retreated cases	876 (8.54)	300 (40.87)	7.404 (6.293–8.712)	<0.001	7.513 (6.372–8.859)	

MDR-TB, multidrug-resistant tuberculosis.

## 4 Discussion

This study represents the first comprehensive analysis of the drug resistance patterns and trends among TB patients in Wenzhou. The findings indicate that the resistance rates of clinical isolates from new patients to FLDs were as follows: INH at 12.15%, SM at 10.89%, RFP at 5.27%, and EMB at 2.71%. In contrast, the resistance rates of clinical isolates from retreated patients to FLDs were significantly higher, with INH at 34.61%, RFP at 27.04%, SM at 23.47%, and EMB at 11.65%. Notably, there were differences in both the order and rates of resistance between the two patient groups regarding FLDs. When compared to the findings of Li et al. (10) in Hangzhou, the resistance order for new patients was inconsistent, whereas it was consistent for retreated patients. Similarly, the resistance order for both new and retreated patients was inconsistent when compared to the results of Song et al. (11) in Shandong. However, in Liu et al.’s study conducted in Lianyungang (12), the resistance order for new and retreated patients was consistent, albeit with variations in resistance rates for FLDs. These differences may be attributed to variations in clinical medication regimens, patient population characteristics, and the genetic background of MTB across different regions. For instance, in the study by Pan et al. (6) in Dalian, a higher proportion of new patients were resistant to isoniazid compared to our study, while our study observed a relatively higher resistance rate to ethambutol among new patients. Such regional differences highlight the importance of understanding local drug resistance patterns to guide clinical treatment and public health interventions. Additionally, the observed differences in drug resistance trends among regions may also reflect the varying effectiveness of TB control strategies and healthcare resource allocation. Future research should further explore the underlying reasons for these regional differences and conduct multicenter studies to provide a more comprehensive understanding of drug resistance trends in TB.

Further analysis revealed that the proportions of DR-TB, PDR-TB, and MDR-TB in newly treated patients was lower than that in retreated patients. This observation can be readily explained by the fact that retreated patients often experience irregular medication adherence or treatment failures, which increases the likelihood of MTB mutating into drug-resistant strains under previous drug pressure. Numerous studies have corroborated this finding (13, 14). Notably, approximately one-tenth of new patients develop MR-TB, which is slightly higher than the proportion observed in retreated patients. Research conducted by Song et al. supports these results (11). The analysis suggests that retreated patients, having undergone multiple drug treatments, are more prone to develop resistance to a broader range of drugs, thereby resulting in a slightly lower proportion of MR-TB. The elevated proportion of MR-TB among new patients, coupled with the higher proportion of MDR-TB in retreated patients, underscores the necessity for medical institutions and disease prevention agencies to enhance the management of the entire treatment process. This approach aims to mitigate the risk of patients requiring retreatment and to implement measures to control the spread of primary DR-TB.

The drug resistance rate of TB patients in Wenzhou City exhibited a general downward trend from 2014 to 2023. This decline is primarily attributed to a significant reduction in the drug resistance rate among retreated patients, which decreased from 64.19% in 2014 to 28.57% in 2023. In contrast, the resistance rate among newly diagnosed patients has remained stable at approximately 18%, showing no downward trend. Research conducted by Pan et al. (6) in Dalian indicated a significant decrease in the drug resistance rate among TB patients from 2013 to 2020, affecting both newly treated and retreated patients. Similarly, a study by Zhang et al. (15) in Chongqing revealed an increase in the drug resistance rate among TB patients in 2019 compared to 2016, for both newly treated and retreated patients. Despite the implementation of the Directly Observed Treatment, Short-course (DOTS) strategy in China since 2001, achieving a coverage rate of 100% by 2006 and witnessing considerable progress in TB control, numerous challenges persist (16). The vast geographical diversity in China, along with disparities in economic development, population density, and healthcare resources, has led to varying impacts of the DOTS strategy across different regions, resulting in divergent trends in TB drug resistance. The results of this study indicate that the control of DR-TB in Wenzhou City has achieved notable progress over the past decade. Notably, among retreated patients, both the number of drug-resistant cases and the rate of drug resistance have demonstrated a downward trend. This finding is of considerable significance. It is widely recognized that primary drug resistance in new TB patients is primarily attributed to transmission, while drug resistance in relapsed patients may arise from inadequate treatment (17–19). Our analysis suggests that the implementation of the DOTS and DOTS-Plus strategies, coupled with government investment in dedicated funds to combat TB, has contributed to a reduction in irregular and inadequately treated cases in Wenzhou, effectively controlling the rate of acquired drug resistance among retreated patients. The drug resistance rate among new patients remains stable, indicating that the transmission of primary DR-TB has not been adequately addressed. According to China’s fifth tuberculosis epidemiological sampling survey report, 43.1% of patients capable of transmitting MTB are asymptomatic, and three-quarters of patients are unaware of their TB status, which inevitably contributes to the transmission of DR-TB (20). Research by Liu et al. (21) indicates that the spread of drug-resistant MTB is the primary driver of DR-TB in China. Therefore, it is imperative for medical and disease prevention and control departments to shift from a strategy of passive discovery to active screening and to implement intervention measures aimed at controlling the spread of primary DR-TB.

The annual trend of drug resistance among new and retreated patients was further stratified and analyzed. Regarding the four FLDs, the resistance rates of SM, RFP, and EMB in new patients exhibited an upward trend, while INH remained relatively stable. In contrast, the resistance rates of the four FLDs in retreated patients showed a significant decrease; however, the resistance rates of SM, INH, and RFP persisted at 20%. Concerning drug resistance patterns, the proportions of PDR-TB and MDR-TB in new patients exhibited an upward trend, while the proportion of MR-TB demonstrated a slight decrease. In retreated patients, the proportions of MR-TB, PDR-TB, and MDR-TB displayed a downward trend, although the resistance rate of MDR-TB remained above 18%. These findings underscore the necessity for the medical department to conduct pDST for both new and retreated patients. Such testing not only informs clinicians on the selection of anti-TB drugs and the formulation of effective treatment plans but also assists disease control departments in understanding local trends in TB resistance, thereby enabling the adjustment of control measures to mitigate the spread of DR-TB (22, 23). This study analyzed the pDST data of TB patients in Wenzhou from 2014 to 2023, demonstrating good consistency and providing a reference for future research on drug resistance trends in the region.

Multivariate analysis revealed that being younger than 65 years, belonging to the Han ethnicity, being part of the migrant population, being unemployed, and having a history of previous treatment are significant risk factors for MDR-TB. Previous studies have also identified individuals younger than 65 years as a risk factor for MDR-TB, likely due to their busier work or study schedules, which may lead to delays in diagnosis and subsequent development of MDR-TB (24, 25). Additionally, Han patients are more predisposed to MDR-TB compared to those from ethnic minority groups. Our analysis indicates that the population of Wenzhou is predominantly Han, with Han patients residing in areas of higher population density and engaging in more frequent social activities, thereby increasing their risk of developing MDR-TB. A systematic analysis by Rajendran et al. (26) on risk factors for MDR-TB in Malaysia similarly identified the Malay population, which constitutes the largest demographic group, as a risk factor for MDR-TB. A meta-analysis focusing on risk factors for MDR-TB in China corroborated our findings, highlighting the migrant population as a significant risk factor (27). Migrant individuals tend to be more mobile and often live in substandard conditions, increasing their susceptibility to MDR-TB. Numerous studies have established a correlation between unemployment and the incidence of MDR-TB (28–30). Our results reinforce the notion that unemployment is a risk factor for MDR-TB; being unemployed typically correlates with low income, which often results in poor living conditions, malnutrition, and prolonged physical labor, all contributing to an elevated risk of developing MDR-TB. Furthermore, individuals with a history of previous treatment may not have eradicated all tuberculosis bacteria due to initial treatment failures or irregular medication adherence, leading to mutations that result in MDR-TB. This association between a history of prior treatment and MDR-TB has been confirmed by various studies (31–33).

This study has several limitations that warrant discussion. First, 18,427 culture-negative cases (accounting for 42% of all registered TB patients) were excluded, which may introduce selection bias and potentially affect the generalizability of the findings. Additionally, as this study was conducted solely in Wenzhou, the regional applicability of its conclusions may be limited. Future research should consider employing more comprehensive diagnostic approaches, such as combining molecular diagnostic techniques with culture methods, to reduce bias caused by diagnostic uncertainty and validate the current findings in broader populations. Second, while this study utilized the liquid pDST system provided by BD, the company does not commercially produce second-line drug susceptibility testing (SLD-DST) reagents, thus preventing routine SLD-DST. This limitation restricted our analysis to FLDs resistance patterns, precluding a comprehensive assessment of the study population’s drug resistance profile, particularly the epidemiological trends of extensively drug-resistant tuberculosis (XDR-TB). As a major global challenge in TB control, XDR-TB exhibits more complex resistance patterns and greater treatment difficulties. The absence of SLD-DST data prevented accurate estimation of XDR-TB prevalence in the study population and direct comparisons with findings from other regions or countries, potentially diminishing the guidance value of this study’s results for XDR-TB prevention strategies. Future studies should routinely perform SLD-DST to provide more comprehensive drug resistance data for clinical decision-making and public health policy formulation. Third, this study did not collect important clinical and demographic information, including smoking status, alcohol consumption history, education level, nutritional status, or comorbidities. These factors may influence treatment outcomes and drug resistance patterns in TB. Subsequent research should incorporate these variables to enable a more comprehensive understanding of TB epidemiology and its influencing factors.

## 5 Conclusion

There are notable differences in drug resistance patterns between patients with new-onset TB and those with a history of TB treatment. From 2014 to 2023, the prevalence of DR-TB in Wenzhou exhibited an overall downward trend, primarily attributable to a decrease in drug resistance among retreated patients. This rate has significantly declined, whereas the drug resistance rate among new TB patients has remained relatively stable. Disease prevention and control agencies should prioritize addressing the spread of primary DR-TB. Additionally, medical institutions should concentrate on high-risk MDR-TB patients who are under 65 years of age, belong to the Han ethnicity, are part of the migrant population, are unemployed, and have a history of prior treatment.

## Data Availability

The raw data supporting the conclusions of this article will be made available by the authors, without undue reservation.
